# Caught Between Vulnerability and Neglect: Nutrition in People with Intellectual Disabilities

**DOI:** 10.3390/nu18020304

**Published:** 2026-01-18

**Authors:** Ellen Margrete Iveland Ersfjord, Helen Kathrine Røstad-Tollefsen, Svein Olav Kolset, Arlene M. McGarty

**Affiliations:** 1 Department of Health and Nursing Science, University of Agder, Jon Lilletuns vei 9, 4879 Grimstad, Norway; 2 Baerum Municipality, Health and Care Services, Community Housing and Services, Postboks 700, 1304 Sandvika, Norway; helen.tollefsen@baerum.kommune.no; 3 Department of Nutrition, Institute of Basic Medical Sciences, University of Oslo, P.O. Box 1046 Blindern, 0317 Oslo, Norway; s.o.kolset@medisin.uio.no; 4 Mental Health and Wellbeing, University of Glasgow, 90 Byres Road, Glasgow G12 8TB, UK; arlene.mcgarty@glasgow.ac.uk

**Keywords:** intellectual disabilities, nutrition, health inequalities

## Abstract

People with intellectual disabilities are disproportionately affected by diet-related health inequalities. This Perspective outlines a dual challenge: (1) intrinsic vulnerabilities—cognitive limitations, health-literacy constraints, and comorbidities—that impair individuals’ ability to make healthy dietary choices, and (2) extrinsic neglect—insufficient support in care environments, inadequate nutrition-related training among informal caregivers and support staff, and structural gaps in policy and services. We argue that this “double jeopardy” undermines nutritional equity and proposes strategies for person-centered nutrition education, caregiver empowerment, supportive food environments, and inclusive policy frameworks. Greater interdisciplinary collaboration and tailored research are urgently needed to ensure nutritional health as a right for people with intellectual disabilities.

## 1. Introduction

The term intellectual disabilities refer to congenital or early-onset, lifelong impairments in cognitive functioning [1] and encompasses a range of conditions, including global developmental delay and Down syndrome [2]. Intellectual disabilities affect approximately ~1–3% of the population [3,4] and can range from mild to profound [5], which will affect their need for support for daily tasks [6]. Although individuals with intellectual disabilities constitute a heterogeneous group, their cognitive impairments affect a range of mental processes essential for learning and reasoning, including attention, memory, problem-solving, language comprehension, decision-making, and the ability to plan and organise information [7]. The ability to think long-term and act accordingly is a major challenge for people with intellectual disabilities. It can therefore be difficult to make good lifestyle choices. For many, this leads to substantial weight gain and a significantly increased risk of adverse health outcomes [6]. People with intellectual disabilities have a higher incidence of diabetes, obesity, inactivity, poorer physical fitness and diet than the general population [8,9]. They have approximately 20 years shorter life expectancy and a higher prevalence of long-term comorbidities [10]. While genetics and metabolic disorders can contribute to this, research shows that an unhealthy lifestyle, poor living environment, and lack of timely access to effective healthcare and preventive actions play significant roles [9,10,11]. It is therefore timely to ask the question: Is the diet-related health of people with intellectual disabilities shaped by an interplay of personal vulnerabilities, such as disability-related barriers to proper nutrition, and systemic neglect within health and care provision, placing them at disproportionate risk for adverse outcomes?

Despite these well-documented diet-related health risks, tailored nutrition education, supportive care environments, and inclusive guidelines for people with intellectual disabilities are systematically lacking [11,12,13]. This article argues that nutritional disadvantages in people with intellectual disabilities arise from a double jeopardy—a combination of intrinsic vulnerability and extrinsic neglect—and proposes a framework for addressing this challenge through policy, practice, and research.

This Perspective advances the literature on nutrition-related health inequalities in people with intellectual disabilities by highlighting that poor diet and nutrition is not only an individual-level behavioral issue, but a systems-level failure to provide accessible and appropriate nutritional support for people with intellectual disabilities. This article combines nutrition and broader lifestyle science, intellectual disabilities research, and public-health perspectives to provide actionable priorities for education, service provision, and policy reform.

## 2. Intrinsic Vulnerability: Nutrition-Making Challenges in People with Intellectual Disabilities

### 2.1. Cognitive and Adaptive Limitations

A core characteristic of intellectual disabilities is reduced conceptual, social, and practical functioning [14]. This can hamper comprehension of nutrition information, portion sizes, meal planning, and food safety. Consequently, people with intellectual disabilities frequently rely on carers or support networks to navigate these challenges effectively. Most people with intellectual disabilities will require some level of support with daily tasks, which may include help with budgeting for food, travel for food shopping, or meal preparation [15]. However, for people with intellectual disabilities, support is not always available at mealtimes, which can limit their ability to purchase or prepare healthy meals and lead to unhealthy dietary choices [16].

### 2.2. Health and Nutrition Literacy Barriers

Nutrition literacy—the ability to obtain, process and understand basic nutrition information for making appropriate dietary decisions—may be reduced in persons with intellectual disabilities [15,16]. Inadequate health literacy is a documented obstacle to healthy behaviors in this group [13,17]. Many existing diet and nutrition focused interventions include cognitively challenging and abstract behaviour change techniques that are not suitable for many people with intellectual disabilities. This limits changes in behaviour, even when people are motivated to change [18]. Without accessible and adapted nutrition information, individuals may lack the foundation to make informed food choices. This is relevant for those able to make such choices, those that are not depend on choices made by carers or family members.

### 2.3. Health Conditions, Comorbidities and Biological Risk Factors

The population with intellectual disabilities shows high prevalence of comorbid conditions, metabolic challenges, and often syndrome-specific health issues, which can affect appetite, nutrient utilization, and risk for obesity or malnutrition [13]. Furthermore, people with intellectual disabilities have a higher prevalence of prescribed medication use, such as antipsychotics [19], which also increase the risk of overweight and obesity, which further highlights the need for tailored nutritional support to balance metabolic risk factors to support a healthy weight. Moreover, malnutrition—including deficiencies in micronutrients and essential fatty acids—has been reported among people with intellectual disabilities, especially where energy intake and dietary variety are limited [15].

Nutrition in people with intellectual disabilities is more complex due to factors and co-morbidities associated with intellectual disabilities. For example, people with intellectual disabilities are more likely to experience challenges with chewing and/or swallowing, have a higher prevalence of gastrointestinal conditions, and sensory sensitivities regarding certain foods, which can all contribute to reduced diet quality and micronutrient adequacy [20]. This demonstrates the heterogeneity of this group, where overweight, obesity, and undernutrition can coexist, and emphasizes the need for tailored nutritional assessment and understanding.

## 3. Extrinsic Vulnerability: Neglect in Support Systems and the Social Environment

### 3.1. Informal Carers and Support-Staff Readiness

Many health and care professionals receive limited training in intellectual disabilities, resulting in insufficient knowledge and preparedness to meet the complex health needs of this population [21,22]. There is also a lack of training for support staff in nutrition, healthy meal planning, dietary needs specific to intellectual disabilities, and strategies to support healthy eating [13,23]. In institutional or community-residence settings, meal preparation is often constrained by time, budget, and staff competence, leading to reliance on convenience or low-quality foods [24]. Moreover, the attitudes and perceived responsibilities of staff influence the extent to which healthy eating is prioritized. Practical cooking skills, applied dietary knowledge, and facilitating healthy diets have been identified as major factors influencing staff’s ability to support residents’ nutrition [23]. A similar lack of knowledge or skills among informal carers will also have a negative impact on people with intellectual disabilities’ nutritional status. Nutritional structures and cultures, such as time constraints, competing priorities, managing risks, and limited resources, all impact the readiness of carers to support nutrition [25]. Therefore, even if carers understand the importance of nutrition, limited autonomy, training, lack of individually defined nutritional plans or institutional barriers may prevent them from applying this knowledge in practice. Change must be implemented at a systems level as opposed to placing the responsibility for change entirely with individual carers.

### 3.2. Environmental, Structural, and Policy Shortcomings

In some countries in Europe, people with intellectual disabilities often live in group homes, supported residences, or rely on others for shopping and food preparation—contexts where food environments are shaped more by institutional routines than by individual preferences. In such settings, research suggests that healthy food access may be limited, meal options standardized and inflexible, and staff resources and nutritional knowledge insufficient [13].

At a policy level, national nutritional guidelines frequently fail to address the specific needs of people with intellectual disabilities. There’s often no requirement for disability-service providers to follow tailored nutritional standards or provide systematic nutrition education [13]. This will also apply to informal carers, who are often responsible for managing the nutritional needs of the person they support [26].

### 3.3. Balancing Rights: Choice and Safety

An important consideration for providing nutritional support is balancing the rights of people with intellectual disabilities who are able to make their own choices regarding their lives and ensure their safety. This is especially important in supported living settings, where a safety focus can cause a more restrictive food environment, resulting in less enjoyment and active participation in nutrition. Food is not only a source of nutrition but a key behaviour that can be pleasurable, encourages social gathering and peer-support, and can have important family and cultural meaning—people with intellectual disabilities should have able to experience all the joys of food beyond basic sustenance. Promoting a rights-based approach could help ensure that both choice and safety are achieved. Supported decision-making could enable people with intellectual disabilities to engage with their own nutrition that also reflects their own preferences, abilities, and wellbeing [27].

## 4. How Vulnerability and Neglect Reinforce Each Other

The combination of disability-specific limitations and environmental neglect produces a vicious cycle. Limited autonomy over food choices—due to disability-related factors and lack of nutrition literacy—becomes compounded by an environment or society that does not compensate for supportive structures. Thus, nutritional disadvantages in intellectual disabilities are not solely a matter of personal choice or behavior—it is a structural inequity rooted in insufficient support systems and policy neglect.

## 5. Implications for Health Promotion and Nutrition Education

### 5.1. Centered, Accessible Nutrition Education

Nutrition education for people with intellectual disabilities must be adapted to be accessible to all. This might include simplified language, visual aids, concrete and step-by-step meal planning tools, and hands-on cooking or grocery-shopping support. Education should aim to build practical daily-life skills rather than only impart abstract knowledge [28]. Evidence from existing interventions highlight that including visual materials, novel learning techniques, and providing opportunities for social participation and connectedness all increase motivation and engagement, which positively impacts nutrition-related behaviors [28]. Consistent implementation of these adaptions requires staff time and increased nutritional competence.

### 5.2. Training and Empowerment of Caregivers

Caregivers—family members, residential staff, disability-support workers—require structured training in nutrition, meal planning, cooking, and motivating healthy eating. This would involve evidence-based curricula, regular refreshers, and possibly inclusion in professional certification for disability services. Caregiver involvement has consistently been shown to be a key mechanism to enable positive behaviour change for people with intellectual disabilities [28]. Empowerment and training of caregivers is therefore a priority. Barriers relating to caregiver time need to be systematically addressed, ensuring clarity on roles and responsibility among the various types of caregivers [12].

### 5.3. Designing Supportive Food Environments

Residential services and community care settings should prioritize access to nutritious, fresh foods; flexible meal plans; and involvement of people with intellectual disabilities in food choice and preparation (to the extent possible). Policy and organizational guidelines should mandate quality standards for food provision in care settings serving people with intellectual disabilities. This will require longer-term planning at organizational and individual levels; for example, ensuring sufficient budget is allocated for purchasing fresh and nutritious food as the higher costs of fresh food often results in people with intellectual disabilities or their caregivers choosing cheaper and less nutritious alternatives [28].

### 5.4. Embedding Nutrition Professionals in Disability Services

Dietitians and nutrition experts should be systematically integrated into disability service organizations to provide assessments, individualized diet plans, staff training, and ongoing follow-up. Collaboration among health professionals, disability-care providers, and social services are essential for sustainable impact. Research evidence demonstrates that the effectiveness of interventions and positive behaviour change is greater when support is delivered by people with the required expertise who can offer tailored, long-term support, as opposed to sporadic advice or support [28]. Embedding nutrition experts into disability services enables more consistent support. This may also overcome existing barriers within care services where high staff turnover and gaps across services can limit the continuity of support [11].

### 5.5. Policy and Research Recommendations

To address this dual challenge, we recommend development of national/regional nutrition guidelines specifically tailored for people with intellectual disabilities. These guidelines should also include information on the effects of different medications on nutrition, as well as strategies to identify, mitigate, and adapt dietary intake accordingly. We also recommend inclusion of nutrition-competency requirements in education and training of carers and certification of disability support staff. Embedding dietetic services are needed into disability care and support frameworks. There is also a great need for conducting intervention research aimed at evaluating accessible nutrition education, supportive meal provision, and environmental changes. Finally, we also recommend including monitoring and evaluating diet quality, health outcomes, and equity in nutrition for people with intellectual disabilities through population health surveillance. This will improve decision making and help target the persistent health inequalities people with intellectual disabilities experience. Current practice(s) in Europe directly conflicts with the United Nations Convention on the Rights of Persons with Disabilities which asserts their right to the highest attainable standard of health [29]. The potential costs of implementing changes needed is likely to be offset by the consequent reduction in healthcare expenditures associated with treating diet-related conditions and comorbidities.

## 6. Conclusions

People with intellectual disabilities face a preventable form of nutritional inequality —a double jeopardy shaped by personal vulnerability and systematic environmental neglect. Addressing this requires more than individual-level education of people with intellectual disabilities: it demands structural changes in how care is organized, policies are formulated, and nutritional support is provided. Ensuring access to a healthy, balanced diet for individuals with intellectual disabilities is not just a matter of public health—it is a matter of rights, dignity, and equity. We also call for the urgent need for multidisciplinary research to generate evidence-based strategies that integrate nutrition, disability care, public health, and policy to reduce inequities and promote the right to a health promoting diet for people with intellectual disabilities.

## Data Availability

Not applicable.
